# Conformational switch of polyglutamine-expanded huntingtin into benign aggregates leads to neuroprotective effect

**DOI:** 10.1038/srep14992

**Published:** 2015-10-09

**Authors:** Chia-Sui Sun, Chi-Chang Lee, Yi-Ni Li, Sunny Yao-Chen Yang, Chih-Hsiang Lin, Yi-Che Chang, Po-Fan Liu, Ruei-Yu He, Chih-Hsien Wang, Wenlung Chen, Yijuang Chern, Joseph Jen-Tse Huang

**Affiliations:** 1 Taiwan International Graduate Program in Molecular Medicine, National Yang-Ming University and Academia Sinica, Taipei, Taiwan; 2 Institute of Chemistry, Academia Sinica, Taipei, Taiwan; 3 Institute of Biomedical Sciences, Academia Sinica, Taipei, Taiwan; 4 Institute of Biochemistry and Molecular Biology, National Yang-Ming University, Taipei, Taiwan; 5 Department of Applied Chemistry, National Chiayi University, Chiayi, Taiwan

## Abstract

The abundant accumulation of inclusion bodies containing polyglutamine-expanded mutant huntingtin (mHTT) aggregates is considered as the key pathological event in Huntington’s disease (HD). Here, we demonstrate that FKBP12, an isomerase that exhibits reduced expression in HD, decreases the amyloidogenicity of mHTT, interrupts its oligomerization process, and structurally promotes the formation of amorphous deposits. By combining fluorescence-activated cell sorting with multiple biophysical techniques, we confirm that FKBP12 reduces the amyloid property of these ultrastructural-distinct mHTT aggregates within cells. Moreover, the neuroprotective effect of FKBP12 is demonstrated in both cellular and nematode models. Finally, we show that FKBP12 also inhibit the fibrillization process of other disease-related and aggregation-prone peptides. Our results suggest a novel function of FKBP12 in ameliorating the proteotoxicity in mHTT, which may shed light on unraveling the roles of FKBP12 in different neurodegenerative diseases and developing possible therapeutic strategies.

The accumulation of soluble protein into insoluble proteinaceous aggregates is considered to be a hallmark feature in various neurodegenerative diseases. This include the misfolding of mutant huntingtin (mHTT) in Huntington’s disease (HD), tau in Alzheimer’s disease (AD), α-synuclein in Parkinson’s disease, and TAR DNA-binding protein 43 (TDP-43) in amyotrophic lateral sclerosis (ALS)^1^,^2^. Despite the heterogeneity in the primary sequences and/or native structures among these pathological proteins and peptides, they all undergo structural transformation to form highly-ordered, fibrillar-shaped, β-sheet-rich amyloid assemblies in the disease state^3^. In general, the aggregation process starts with initial conformational alteration from the monomeric protein, following by trapping of monomers into transient populated intermediates, including oligomers and/or protofibers. Through the nucleation and propagation process, the intermediates will subsequently self-assemble into amyloid or amyloid-like fibrils, which sometimes lead to cytotoxicity and/or neurodegeneration^4^.

In HD, many pathomechanisms have been proposed based on the high aggregation propensity of polyglutamine (polyQ) expansions, which is evidenced by striatal atrophy accompanied by the presence of massive intracellular aggregates comprising mHTT N-terminal fragments^1^,^3^,^5^. It has been shown that the expansion of polyQ (>35–40Q) within Htt exon 1 triggers the protein misfolding/aggregation process, which results in the formation of antiparallel-β-rich and detergent-resistant filamentous aggregates that share many characteristics with amyloid fibrils^6^,^7^. During HD pathogenesis, neuronal death within the brain occurs first within the striatum, where it is also most severe^8^,^9^. A previous report has shown that the FKBP12 protein, a peptidyl-prolyl *cis/trans* isomerase and a member of the FK506-binding protein (FKBP) family, is markedly reduced in the striatum of HD mice^10^. Expression of FKBP12 was also found to be decreased in the brain of AD patients and the anterior horn neurons of patients with motor neuron diseases (MND), suggesting that the loss of FKBP12 may contribute to the pathogenesis of HD, as well as other neurodegenerative diseases^11^,^12^. However, the role of FKBP12 in neurodegeneration and its contribution to aggregation and neurotoxicity have not been well characterized.

Various biochemical and cell-biological studies have shown that polyQ repeats are able to fold into divergent conformations under different conditions, resulting in the existence of multiple aggregation pathways^13^. Nekooki-Machida and colleagues previously demonstrated that structurally-distinct amyloid conformers of polyQ-expanded mHTT displayed different cytotoxicities^14^. Furthermore, the secondary structures of inclusions were shown to associate with the severity of the degenerated brain region in both mice and patients with HD, suggesting a close relationship between the amyloid conformation and cellular vulnerability in the affected area^14^,^15^. In the aforementioned cases, the perturbation of the mHTT folding/misfolding process is correlated with aggregate structure, which affects cytotoxicity. Based on the observations that FKBP12 is involved in protein folding/misfolding^16^ and that FKBP12 expression is reduced in HD^10^,^11^, we hypothesized that FKBP12 may modulate the misfolding of mHTT to perturb its proteinopathy.

Here, we employed multiple biophysical/chemical approaches to characterize the conformational change of mHTT aggregates in the presence or absence of FKBP12, using both the recombinant glutathione S-transferase (GST)-mHTT fusion protein and cellular-expressed mHTT-eYFP. Fluorescence-activated cell sorting (FACS) was utilized to harvest mHTT-eYFP aggregates from Neuro2a (N2A) cells for further characterization of their amyloid and structural properties. The beneficial effect of FKBP12 in N2A cells and *C. elegans* as well as its modulation of the polyQ-mediated oligomerization process were also examined. In addition, we explored the potential role of FKBP12 toward other disease-related (polyQ diseases and ALS) and aggregation-prone polypeptides. Our results demonstrate FKBP12 affects both the structural and proteotoxic properties of mHTT aggregates, suggesting the novel function of FKBP12 in HD as well as its potential ability in inhibiting the fibrillogenesis process in other neurodegenerative diseases.

## Results

### FKBP12 decreases the amyloidogenicity of mHTT and structurally promotes the formation of amorphous deposits *in vitro*

**Figure 1:**
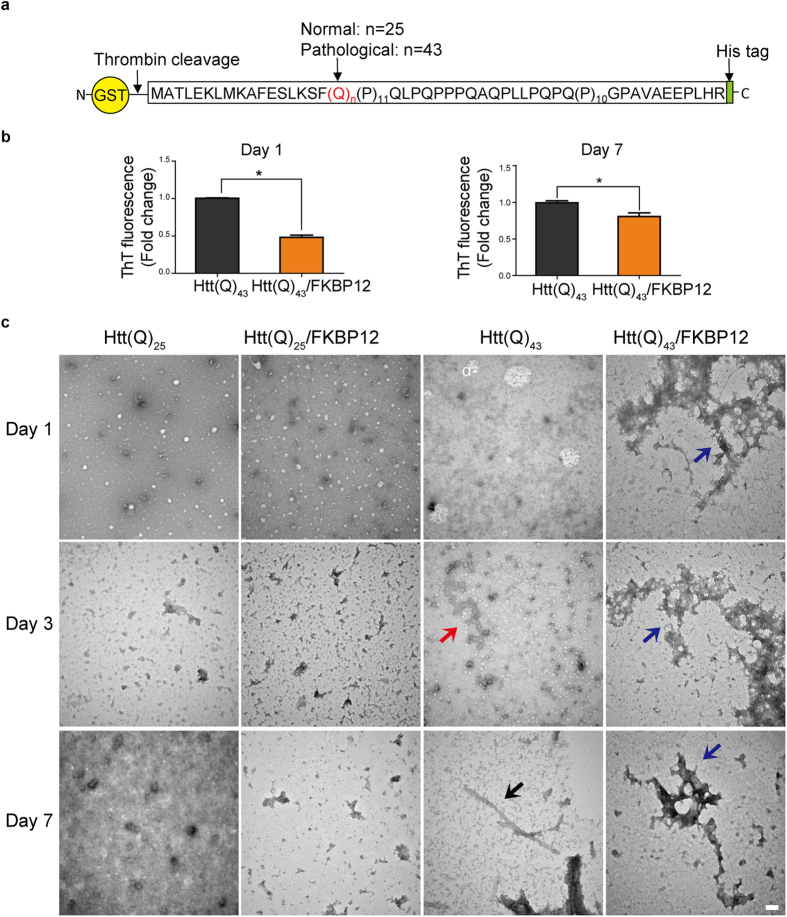
FKBP12 decreases the amyloidogenicity of Htt(Q)_43_ and promotes the formation of amorphous aggregates. (**a**) A schematic representation of the GST-tagged constructs expressing Huntingtin exon 1 with 25 or 43 polyglutamine tracts. (**b**) The fold change in ThT fluorescence between Htt(Q)_43_ and Htt(Q)_43_/FKBP12 after GST removal, on days 1 and 7. All data are presented as means with SEM (n = 3). *p < 0.05, Student’s *t*-test (**c**) TEM images of the Htt(Q)_25_ and Htt(Q)_43_ proteins in the presence/absence of FKBP12 after GST removal. Morphological changes of the Htt(Q)_43_ proteins are depicted as follows: red arrow, protofibrils; black arrow, fibril; blue arrow, amorphous aggregates. Scale bars represent 100 nm.

To determine whether FKBP12 can directly modulate mHTT-mediated amyloidogenicity, we purified GST-Htt(Q)_n_ proteins, consisting of huntingtin (Htt) exon 1 with 25 or 43 polyglutamine repeats fused to glutathione S-transferase (GST) (Fig. 1a). Htt exon 1 was chosen as its expression promotes the development of clinical symptoms resembling the pathological features of HD^17^,^18^. The purity and biochemical properties of all the aforementioned recombinant proteins are characterized by SDS-PAGE and Western blot (Supplementary Fig. 1). In addition, Htt(Q)_43_ exhibited higher Thioflavin T (ThT) fluorescence than Htt(Q)_25_ after thrombin digestion in the ThT assay (Supplementary Fig. 2a,b), suggesting that polyQ-expanded mHTT possesses substantial amyloidogenicity. An equimolar ratio of FKBP12 was co-incubated with GST-Htt(Q)_43_ and the co-incubation groups are henceforth referred to as Htt(Q)_43_/FKBP12. Compared with Htt(Q)_43_ alone, ThT fluorescence significantly reduced in Htt(Q)_43_/FKBP12 on both day 1 (~50% reduction) and 7 (~20% reduction) (Fig. 1b).

Examination of protein ultrastructure by transmission electron microscopy (TEM) analysis revealed a heterogeneous population of protofibrils and their corresponding assemblies for Htt(Q)_43_ (Fig. 1c, red arrow) after 3 days of incubation, which are considered to be the precursor of polyglutamine fibrillization. Linear fibers, approximately 13 nm in width, were present in Htt(Q)_43_ samples at day 7 (Fig. 1c, black arrow), while no such structures were observed for Htt(Q)_25_ (Fig. 1c, left column). Note that the presence of FKBP12 inhibited mHTT fibrillization and strongly promoted the formation of amorphous assemblies, as observed for Htt(Q)_43_/FKBP12 on days 1, 3, and 7 (Fig. 1c, blue arrow). The addition of FKBP12 had a negligible effect on the aggregates in Htt(Q)_25_/FKBP12 (Fig. 1c, second column). Our results suggest that FKBP12 is able to perturb the assembly of Htt(Q)_43_ *in vitro*, which leads to a decrease in amyloid fiber formation.

### FKBP12 reduces the amyloid property and changes the ultrastructure of mHTT inclusions in N2A cells

**Figure 2:**
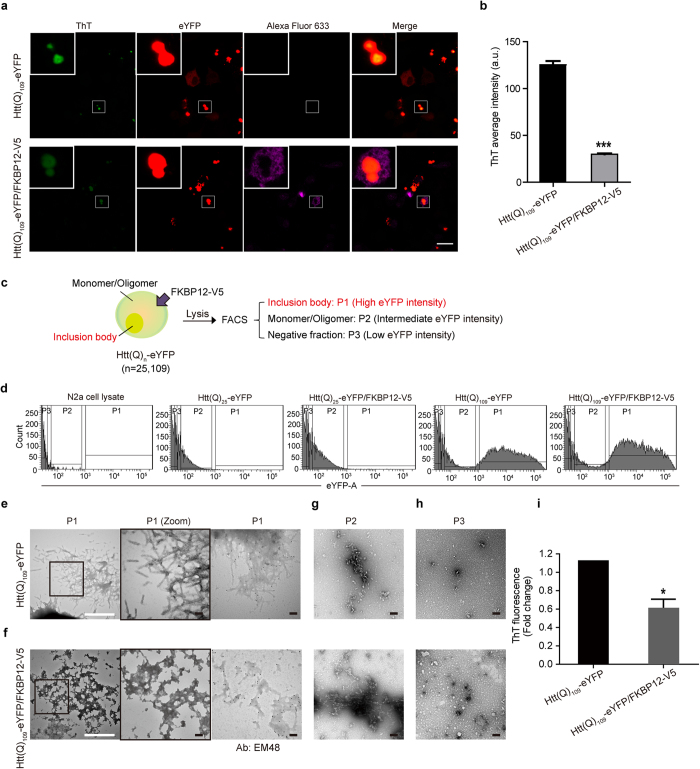
FKBP12 reduces the amyloid property of mHTT in N2A-expressing cells. (**a**) Whole-cell staining of Htt(Q)_109_-eYFP and Htt(Q)_109_-eYFP/FKBP12-V5 transfectants using ThT dye. Note that the inclusions of Htt(Q)_109_-eYFP and Htt(Q)_109_-eYFP/FKBP12-V5 were co-localized with ThT signal. Insets indicate the enlarged ThT signal. Both Htt(Q)_109_-eYFP and Htt(Q)_109_-eYFP/FKBP12-V5 were immunostained with anti-V5 followed by probing with Alexa Fluor 633 anti-mouse Ab. Scale bar: 20 μm. (**b**) Fold change in ThT fluorescence between the Htt(Q)_109_-eYFP and Htt(Q)_109_-eYFP/FKBP12 groups. (**c**) Representative diagram of the workflow using FACS to separate the lysates of cells co-expressing Htt(Q)_n_-eYFP (n = 25, 109) and FKBP12 into three fractions (P1, P2, and P3) based on their eYFP fluorescence intensity. (**d**) Sorting histograms of N2A cell lysate, Htt(Q)_25_-eYFP, Htt(Q)_25_-eYFP/FKBP12-V5, Htt(Q)_109_-eYFP, and Htt(Q)_109_-eYFP/FKBP12-V5. (**e**,**f**) TEM micrographs of the P1 fraction from Htt(Q)_109_-eYFP and Htt(Q)_109_-eYFP/FKBP12-V5. Middle graphs are enlarged images of the black square in the corresponding left panel. Right graphs indicate the immunolabeling of the sorted particles in Htt(Q)_109_-eYFP and Htt(Q)_109_-eYFP/FKBP12 samples with EM48 antibody. White and black scale bars represent 500 and 100 nm, respectively. (**g**) Images of the sorted particles from the P2 fraction. (**h**) Images of the sorted materials from the P3 fraction. (i) Fold change in ThT of the collected aggregates from P1 between the Htt(Q)_109_-eYFP and Htt(Q)_109_-eYFP/FKBP12 groups. n = 3, *p < 0.05, as analyzed by Student’s *t*-test.

Although we have established that co-incubation of recombinant FKBP12 with thrombin-released mHTT resulted in conformational change *in vitro*, the structural and biological impact of FKBP12 in mHTT-expressing mammalian cells remains enigmatic. To determine whether FKBP12 affects the amyloid property of mHTT aggregates within the cell, we constructed plasmids encoding pathological (n = 109) polyQ tracts fused with enhanced yellow fluorescent protein (eYFP) (Htt(Q)_109_-eYFP), and co-transfected N2a cells with these plasmids and FKBP12-V5 or vector. Inclusion bodies of Htt(Q)_109_-eYFP-expressing cells colocalized with strong ThT fluorescence signal, indicating their strong amyloid property. For cells co-expressing Htt(Q)_109_-eYFP and FKBP12-V5 (referred as Htt(Q)_109_-eYFP/FKBP12-V5), inclusions colocalized with faint ThT signal (Fig. 2a), showing the coexpression of FKBP12 altered the amyloidogenicity in cells. In addition, we also observed the diffused and peripheral localization of FKBP12-V5 (probed with Alexa Fluor 633) around Htt(Q)_109_-eYFP inclusions, which suggests FKBP12 does not coaggregate with mHTT inclusions (Fig. 2a). Quantitative analysis revealed a significant ~75% reduction of ThT in Htt(Q)_109_-eYFP/FKBP12-V5 as compared to Htt(Q)_109_-eYFP (Fig. 2b).

While the ThT staining displayed the changes in overall amyloid properties within the cell, it is unable to provide detailed ultrastructural information on the mHTT inclusions modulated by FKBP12. Due to the low solubility and complexity of the inclusion bodies, it is difficult to purify the mHTT aggregates from N2A cells. We therefore employed fluorescence-activated cell sorting (FACS) to collect and analyze inclusion bodies (P1), monomer/oligomer mixtures (P2), and cell debris (P3) from cell lysates containing Htt(Q)_25_-eYFP, Htt(Q)_25_-eYFP/FKBP12-V5, Htt(Q)_109_-eYFP, and Htt(Q)_109_-eYFP/FKBP12-V5 (Fig. 2c,d). FACS enables the purification and accumulation of HTT-specific particles in a high-throughput manner for TEM and ThT analysis. The gating strategy for collection of different fractions (P1-3) was based on their eYFP florescence intensity, as reported in an earlier study^19^. The sorted materials from the P1 fraction exhibited over 99% purity for both Htt(Q)_109_-eYFP and Htt(Q)_109_-eYFP/FKBP12-V5 (Supplementary Fig. 3a,b). The P1 fraction from Htt(Q)_109_-eYFP alone possessed a fibrillar structure, as demonstrated by TEM (Fig. 2e, left and middle panels). Importantly, co-expression of FKBP12-V5 and Htt(Q)_109_-eYFP mainly resulted in amorphous aggregates in the same fraction (Fig. 2f, left and middle panels). Immunogold labeling using EM48 antibody against the N-terminus of Huntingtin protein further confirmed the observed structures from both transfectants were HTT-specific species (Fig. 2e,f, right panels). In addition, we observed protofibril-like/oligomers in the P2 fraction, and demonstrated that FKBP12 has negligible impact on mHTT morphology in this fraction (Fig. 2g). As regards the P3 fraction, we suspect it was mainly comprised of cell debris due to its low eYFP signal (Fig. 2h).

To further assess whether the amyloid propensity of mHTT inclusions are modulated by the presence of FKBP12, we subjected the sorted materials to ThT assays. ThT fluorescence in the P1 fraction of the Htt(Q)_109_-eYFP/FKBP12-V5 group was reduced by approximately 45% compared with the Htt(Q)_109_-eYFP group (Fig. 2i). Collectively, these data suggest that FKBP12 shifts the aggregation process by inhibiting the formation of amyloidogenic fibrils and promoting the non-amyloidogenic/amorphous aggregates production in N2A cells.

### FKBP12 interacts with mHTT and modifies the biochemical and physiochemical properties of mHTT oligomers

**Figure 3:**
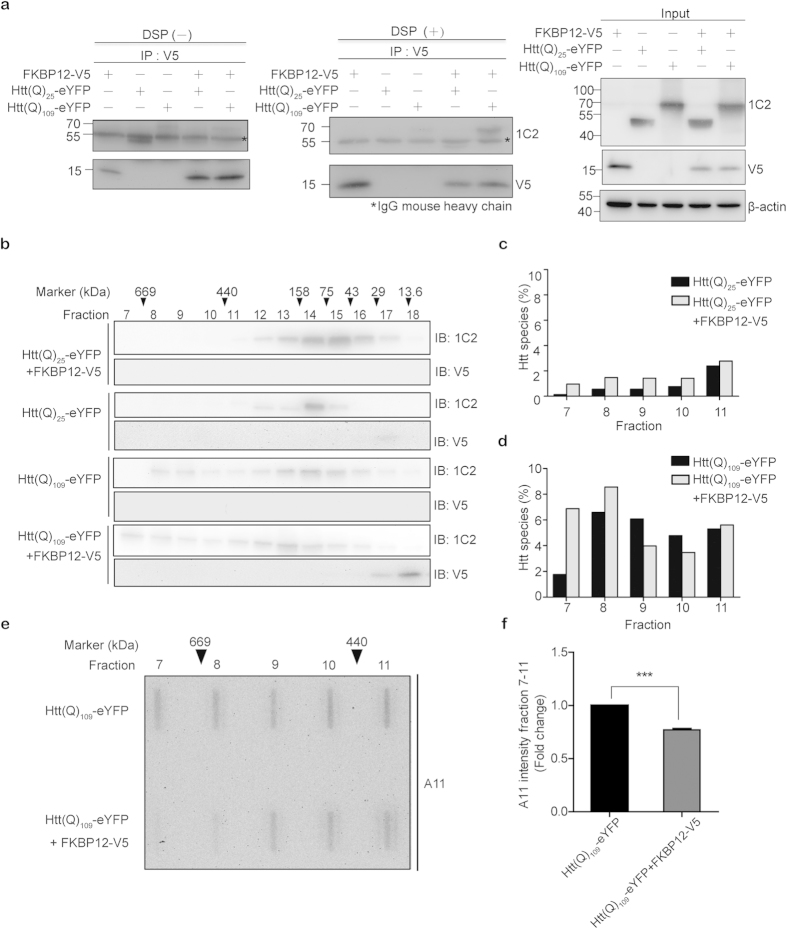
FKBP12 interacts with mHTT and increases higher-order and low A11-reactive oligomeric mHTT species in N2A cells. (**a**) Htt(Q)_25_-eYFP and Htt(Q)_109_-eYFP (co-expressed with FKBP12 or vector alone) were immunoprecipitated (IP) with V5 antibody, and probed with 1C2 antibody in the absence/presence of DSP (left and middle panels). Western blot was performed to examine expression of Htt(Q)_25_-eYFP, Htt(Q)_109_-eYFP, and FKBP12-V5 using the indicated antibodies (right panel). (**b**) Cell lysates from each transfectants were fractionated using size exclusion column to determine the sizing behavior of HTT proteins. Each column fraction was analyzed by SDS-PAGE/Western blot with 1C2 or V5 antibodies. The positions of the molecular mass standards are depicted using arrowheads. (**c**,**d**) Fractions 7–11 of the Htt(Q)_25_-eYFP, Htt(Q)_25_-eYFP/FKBP12, Htt(Q)_109_-eYFP, and Htt(Q)_109_-eYFP/FKBP12 samples were probed with 1C2, and quantified using Image J. (**e**,**f**) Non-denatured fractions 7–11 from Htt(Q)_109_-eYFP and Htt(Q)_109_-eYFP/FKBP12 samples were applied onto a slot-blot apparatus, probed with anti-oligomer A11 antibody, and quantified using Image J. Bars represent means ± SEM (n = 4). ***p < 0.001, as analyzed by Student’s *t*-test.

To evaluate the potential interaction between FKBP12 and HTT proteins within N2A cells, we performed *in vivo* cross-linking experiments. A membrane-permeable, thiol-cleavable crosslinker, dithiobis(succinimidyl propionate) (DSP), was added to the medium to stabilize the putative interactions within the cell between FKBP12 and Htt(Q)_25_-eYFP or Htt(Q)_109_-eYFP. DSP is an effective, reversible crosslinker with a 12 Å spacer arm, which utilizes its amine-reactive ester groups to react with primary amines such as lysine or the N-terminus of proteins^20^. After introducing DSP, cell lysates were immunoprecipitated with anti-V5, and probed with the antibody which recognized the polyglutamine tract, 1C2. Co-immunoprecipitation (co-IP) revealed that Htt(Q)_109_-eYFP, but not Htt(Q)_25_-eYFP, interacted with FKBP12 (Fig. 3a, middle panel), which hints at a selective affinity of FKBP12 for mHTT. We failed to detect an interaction between FKBP12 and Htt(Q)_109_-eYFP in the absence of DSP, which suggests that the interaction between these two proteins is transient and weak (Fig. 3a, left panel).

Having confirmed the *in vivo* interaction by co-IP, we further investigated the biochemical and physiochemical effects of FKBP12 on mHTT. N2A cells were co-transfected with Htt(Q)_n_-eYFP (n = 25, 109) constructs and FKBP12-V5 or empty vector to determine the capability of FKBP12 to modulate the aggregation propensity of mHTT. While negligible amounts of the aggregate were retained on the filter membrane in Htt(Q)_25_-eYFP lysate, large amounts of EM48-positive deposits were detected in Htt(Q)_109_-eYFP lysate by the filter retardation assay. To our surprise, expression of FKBP12 did not influence the level of Htt(Q)_109_-eYFP aggregates (Supplementary Fig. 4a). This phenomenon was confirmed by the observation that expression of either Htt(Q)_109_-eYFP or Htt(Q)_109_-eYFP/FKBP12-V5 resulted in the retention of abundant SDS-insoluble, high molecular weight (MW) aggregates in the stacking gel (Supplementary Fig. 4b). In addition, the soluble portion of Htt(Q)_25_-eYFP and Htt(Q)_109_-eYFP was also unaffected by co-expression with FKBP12, as apparent in the resolving gel. Moreover, the presence of FKBP12 did not change the localization of Htt(Q)_25_-eYFP and Htt(Q)_109_-eYFP in the cell, as observed by confocal microscopy (Supplementary Fig. 5). Collectively, these results indicate that the total amount of mHTT in both the soluble and insoluble fractions is unaffected by FKBP12.

We next assessed whether the oligomerization process and biochemical properties of mHTT oligomers are affected by FKBP12. To separate oligomers according to their molecular weight (MW), we used size exclusion chromatography to fractionate lysates from cells expressing Htt(Q)_n_-eYFP (n = 25 or 109) in the presence or absence of FKBP12. Each fraction was then subjected to Western blot analysis with an anti-polyglutamine antibody, 1C2, to reveal the distribution of HTT oligomers. High-MW HTT oligomeric species from Htt(Q)_109_-eYFP were mostly detected in fractions 8-10 (~440–669 kDa), whereas the presence of FKBP12 shifted oligomers to fractions 7–8 (>669 kDa) (Fig. 3b,d). In contrast, these high-MW species were rarely observed in the equivalent fractions of their Htt(Q)_25_-eYFP or Htt(Q)_25_-eYFP/FKBP12–V5 counterparts (Fig. 3b,c). Interestingly, fractions 7 and 8 of Htt(Q)_109_-eYFP/FKBP12–V5 were not recognized by the amyloid oligomer-specific antibody, A11 (Fig. 3e). Previous studies have shown that oligomers arrested in A11-immunoreactive conformers are more hydrophobic and proteotoxic to neuroblastoma cells^21^,^22^. The quantitative analysis of the A11 immunoreactive signals showed significant reduction in high-MW HTT oligomers (fraction 7–10) (Fig. 3f), suggesting a possible conformational rearrangement in the oligomers. Our finding that mHTT was redistributed from high-MW oligomer pools to A11-negative conformers suggest FKBP12 remodeled the assembly of mHTT oligomeric intermediates to non-proteotoxic species. These oligomers, distinct in their conformation and proteotoxicity, may further reduce downstream aberrant protein-protein interactions, and exert beneficiary effects.

### FKBP12 ameliorates polyQ-mediated neurotoxicity in both N2A cells and *C. elegans*

**Figure 4:**
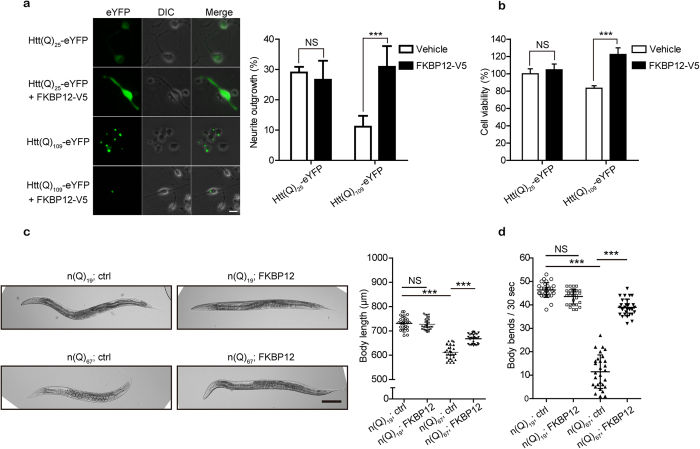
FKBP12 confers neuroprotection against polyQ-mediated neurotoxicity in mHTT-expressing N2A cells and *C. elegans.* (**a**) The neurite outgrowth of cells co-expressing Htt(Q)_25_-eYFP or Htt(Q)_109_-eYFP with FKBP12 or empty vector. Note that only HTT-expressing cells, which exhibited eYFP fluorescence, were chosen for analysis. (**b**) The effect of FKBP12 on the viability of mHTT-expressing N2A cells was examined using the AlamarBlue assay. (**c**) Panel left: The DIC micrographs of 4-day-old n(Q)_19_ and n(Q)_67_ animals co-expressing FKBP12 or control vector [referred as n(Q)_19_;FKBP12, n(Q)_19_;Ctrl, n(Q)_67_;FKBP12, and n(Q)_67_;ctrl, respectively]. Scale bar represents 100 μm. Panel right: Quantitative analysis of the body length among the transgenic *C. elegans* lines. (n = 30, Bars represent means ± standard deviation) (**d**) The motility assay was determined by the number of body bends within 30 seconds in various *C. elegans* lines. (For all transgenic lines, n = 29) Statistical significance was calculated by one-way analysis of variance followed by posthoc Tukey’s test (***p < 0.001, NS: Not significant).

To investigate the biological impact of FKBP12 in polyQ-expanded HTT-expressing cells, we proceeded to subject cells co-expressing FKBP12 and Htt(Q)_25_-eYFP or Htt(Q)_109_-eYFP to retinoic acid (RA)-induced neurite outgrowth assays. Only eYFP-positive cells with intact nuclei and normal shape/volume were chosen for quantification. Further, of the eYFP-positive N2A cells, only cells with processes twice as long as the cell body were counted as neurite-positive cells (details in the Materials and Methods section). The presence of FKBP12 enhanced the proportion of mHTT-expressing cells that were neurite-positive (from 11% to 31%) after 48 hours of RA treatment (Fig. 4a). To further examine whether FKBP12 restores the viability of cells expressing mHTT, Htt(Q)_25_-eYFP and Htt(Q)_109_-eYFP were co-expressed with either FKBP12 or vector alone in N2A for 48 hours. Since increased oxidative stress is commonly observed in HD patients^23^, H_2_O_2_ was added to media for 3 hours prior to our viability analysis (details in the Materials and Methods section); the viability of cells expressing Htt(Q)_109_-eYFP was decreased by about 20% when compared to those expressing Htt(Q)_25_-eYFP. In addition, while FKPB12 had no effect on cells expressing Htt(Q)_25_-eYFP, it significantly increased the viability of cells expressing Htt(Q)_109_-eYFP (Fig. 4b). Taken together, these results indicate that FKBP12 exerts neuroprotective effects toward mHTT-expressing cells.

Previously, the ectopic expression of expanded-polyQ in nervous system of nematode, *Caenorhabditis elegans,* has been implicated to neuronal dysfunction and served as an excellent platform in monitoring the proteotoxicity associated with the expressed polyQ protein^24^. Taking advantage of the simple but compact nerve system in *C. elegans* as well as its clear linkage between behavior and function with particular neurons, we sought efforts to learn whether FKBP12 also confers neuroprotective effect in the organismal level. We established a pan-neuronal expressing FKBP12 in *C. elegans* and examined its beneficial effect on the polyQ disease model. The extrachromosomal arrays that ectopically express FKBP12 as well as control plasmid were observed across *C. elegans* nervous system and crossed into two different polyQ expressing backgrounds, including 19 and 67 polyQ repeats, respectively (details in Material and Methods). The neuronal expression of 19 polyQ repeats fused with cyan fluorescent protein (CFP) in *C. elegans* [denoted as n(Q)_19_;ctrl] exhibited diffuse, soluble distribution pattern in the neuronal processes and the cell bodies (Supplementary Fig. 6). Conversely, 67 polyQ repeats fused with CFP [denoted as n(Q)_67_;ctrl] developed discrete foci, indicating aggregate formation in the neurons. Notably, the co-expression of the FKBP12 in 67Q-expressing animals [denoted as n(Q)_67_;FKBP12] did not affect the apparent aggregate formation in C. *elegans*, which is consistent with the observation in the N2A model.

While n(Q)_67_;ctrl is smaller compared with n(Q)_19_;ctrl in their body size as also described in literature^24^, n(Q)_67_;FKBP12 animals displayed significant improvement in the body size (Fig. 4c). We further examined the motility of the transgenic strains since the dysfunction or loss of neuronal cells has shown to cause uncoordination or paralysis in *C. elegans*^25^. Reduced motility was observed in n(Q)_67_;ctrl group compared with n(Q)_19_;ctrl, indicating severe neuronal dysfunction in 67Q-expressing animals. Strikingly, behavioral dysfunction was significantly improved in n(Q)_67_;FKBP12 (Fig. 4d and Supplementary video). Provided that such neurotoxicity is considered as a systemic stress response^26^, our findings in the improvement of mobility and body size suggests FKBP12 genetically rescues polyQ-mediated neuronal dysfunction in *C. elegans.*

### FKBP12 may structurally promote other disease-related and aggregation-prone peptides to form amorphous deposits

**Figure 5:**
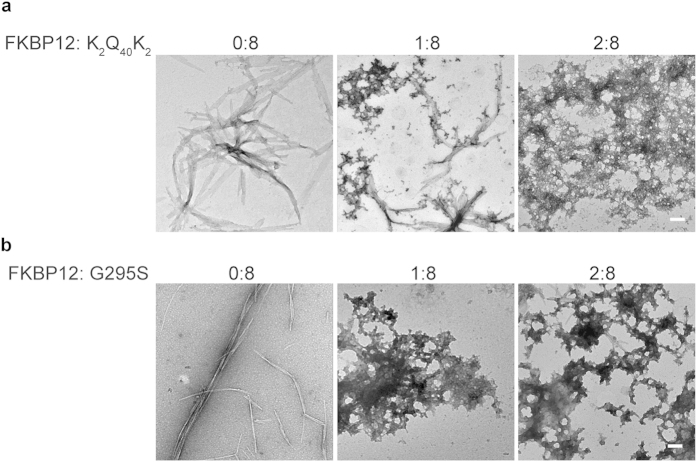
FKBP12 promotes the formation of amorphous aggregates of K_2_Q_40_K_2_ and G295S peptides. (**a**,**b**) TEM images of K_2_Q_40_K_2_ or G295S peptides co-incubated with the indicated molar ratios of FKBP12 in PBS at 37 °C for 7 days. Scale bars represent 100 nm.

We further explored whether FKBP12 is able to structurally-modulate the conformation of other disease-related and aggregation-prone peptides. A chemically-synthesized polyQ peptide (K_2_Q_40_K_2_) was chosen as the polyglutamine tract is believed to be the culprit not only in HD but also in other polyglutamine diseases, such as spinocerebellar ataxias and spinal bulbar muscular atrophy^27^. Previous reports showed K_2_Q_40_K_2_ peptide exhibited high aggregation propensity, formed amyloid-like aggregates, and jeopardized cell viability^28^. Similarly, we found that the synthetic K_2_Q_40_K_2_ formed large, ordered fibrils with ribbon morphology after 7 days of incubation (Fig. 5, upper left panel). Notably, co-incubation of FKBP12 with K_2_Q_40_K_2_ (FKBP12:K_2_Q_40_K_2_ = 1:8) resulted in heterogeneous population of amorphous aggregates and fibrils (Fig. 5, upper middle panel). Formation of abundant amorphous aggregates was observed when we increased the amount of FKBP12 (FKBP12:K_2_Q_40_K_2_ = 2:8) (Fig. 5, upper right panel). In addition, another disease-related peptide, G295S, was also applied to see whether a similar phenomenon can be observed. G295S is a TDP-43 C-terminal fragment (residues 287–322) that possesses an ALS-related mutation and displays the favorability to form β-amyloid, cause membrane leakage, and induces cell death^29^. Our results demonstrate co-incubation of FKBP12 with G295S also results in accumulation of amorphous aggregates and reduced ThT fluorescence intensity (lower panel in Fig. 5 and Supplementary Fig. 7), hinting on the potential role of FKBP12 to shift the aggregation process of amyloid-prone protein to form benign aggregates in different neurodegenerative diseases.

## Discussion

While the accumulation of polyglutamine aggregates is a key pathological event in HD, there is considerable debate as to whether the aggregates induce cytotoxicity. Many studies have demonstrated that the presence of aggregates may lead to neurodegeneration^30^, whereas others suggested that aggregation formation serves as a protective mechanism to reduce soluble β-sheet monomers/oligomers, and thus alleviate cytotoxicity^31^,^32^. This discrepancy is probably due to the diverse, conformational-distinct, structural polymorphisms of amyloids, which result in various pathological phenotypes^33^,^34^. Similar phenomena have been observed for mHTT, Aβ, and Tau, with the formation of distinct conformations during amyloid formation further influencing pathophysiology^14^,^35^,^36^,^37^. Recently, it has been shown that the use of mutant peptides or small molecule inhibitors to manipulate the conformational conversion of TDP-43, Aβ, and α-synuclein during amyloidogenesis reduces the proteotoxicity of these pathological proteins^29^,^38^.

To determine whether FKBP12 is able to reduce the amyloid properties of mHTT aggregates in a cellular system, we utilized amyloid staining dye (ThT) to examine mHTT aggregates co-expressed with FKBP12. Whole-cell ThT staining provides a fast screening system with which to assess the overall amyloid property in the cell (Fig. 2a). However, due to the crowded and heterogeneous environment in the N2A cell, it is still possible that some off-target ThT staining may occur. Thus, we combined fluorescence-activated cell sorting (FACS) with ThT staining and TEM techniques to interrogate the effects of FKBP12 on the biochemical properties and ultrastructural transformation of mHTT inclusions. Cell sorters are advantageous in that they provide a high-throughput platform with which to exclude the unwanted cellular endogenous proteins, and to specifically isolate mHTT-eYFP inclusions with high purity and homogeneity^19^. Hence, our findings using FACS/ThT staining can directly reflect the amyloid properties of the mHTT inclusions, rather than being biased by non-specific binding to other cellular proteins. The filamentous deposits of Htt(Q)_109_-eYFP inclusions isolated by cell sorter (Fig. 2e) resemble the *in vivo* ultrastructure of fibrillar aggregates found in the tissue section of HD-post mortem brains and HD mouse models^30^. Moreover, immunogold-TEM results further confirmed the isolated fibrous deposits arise from mHTT itself, suggesting the feasibility of obtaining eYFP-gated aggregates by FACS. Through an identical gating procedure, amorphous inclusions purified from the Htt(Q)_109_-eYFP/FKBP12 group were identified as distinct aggregates with morphologies and amyloid properties different to those of Htt(Q)_109_-eYFP. It has been previously indicated that molecular chaperones, proteasomes, and other relative factors are sequestered into mHTT aggregates upon HD progression^39^, which may lead to impaired protein folding and degradation, and accumulation of toxic mHTT oligomers. Benign mHTT aggregates (amorphous structure) may serve as a cellular protective response to mitigate the increase of toxic oligomers^40^. We thus suggested that the formation of mHTT aggregates can either be amyloidogenic or non-amyloidogenic, and this process can be mediated in the presence of FKBP12.

FKBP12 has also been reported to participate in various cellular processes, including the maintenance of intracellular Ca^2+^ homeostasis through interaction with the ryanodine receptor (RyR1), cellular signaling, protein folding, the cell cycle, and protein trafficking^41^. Since FKBP12 (i) has been recognized to interact with diverse range of partners and (ii) is required in large quantities for catalysis, it is speculated that a reduction of FKBP12 would lead to misfolding and/or abnormal proteostasis of the target proteins. The pathophysiological role of FKBP12 in HD further caught our attention, as a significant reduction of FKBP12 during disease progression was observed in the striatum of a HD mouse model (R6/2)^10^. In fact, it has been reported that the level of FKBP12 in the striatum of the brain is much higher than in any peripheral tissues^12^, suggesting the degeneration of this protein may play a critical role in HD pathogenesis^10^.

**Figure 6:**
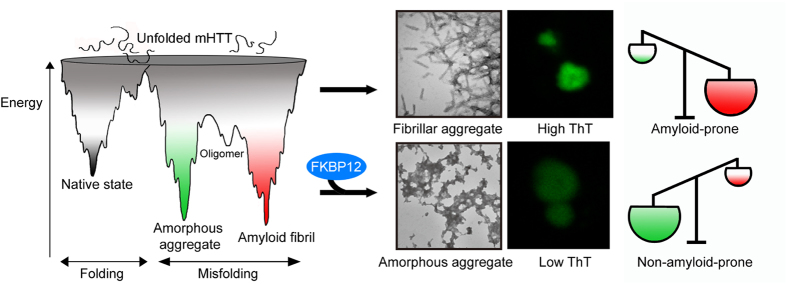
A schematic representation of the structural modulation of mHTT by FKBP12. Through intramolecular contacts, the conformation of mHTT may form into either amyloid fibrils (red) or amorphous aggregates (green). During the aggregation process, the population of mHTT aggregates is prone to form fibrillar-shaped, high ThT amyloid deposits (red). However, in the presence of FKBP12, the free energy landscape of mHTT is reshaped to favor the formation of benign amorphous aggregates with low ThT (green).

Recent studies have indicated that prolyl *cis-trans* isomerization by PPIase may act as a fundamental molecular timer in regulating human physiology and pathology^42^. In this study, we also tried to uncover the possible mechanism of how FKBP12, a known PPIase protein, induces the benign/amorphous aggregates of mHTT in HD. Despite mHTT possess a polyproline domain which can be a potential substrate for PPIase, the aggregation process is also perturbed in the peptide without any proline (K_2_Q_40_K_2_) *in vitro*. Moreover, the suppressed ThT intensity can still be observed when either FK506 or Rapamycin, the PPIase inhibitor, was added into the GST-Htt(Q)_43_ protein co-incubated with FKBP12 (referred as GST-Htt(Q)_43_/FKBP12/FK506 or GST-Htt(Q)_43_/FKBP12/Rapamycin) (Supplementary Fig. 8a). These findings imply the suppression of mHTT amyloidogenicity does not result from the PPIase activity of FKBP12. Also, we observed that FKBP12 does not coaggregate with mHTT cellular insoluble aggregates (Supplementary Fig. 8b; Fig. 2a). Similar phenomena can also be confirmed in the GST-Htt(Q)_43_ system in the presence of FKBP12 (data not shown). Our results suggest that FKBP12 may undergo a “hit-and-run” action mode with soluble mHTT and ameliorates mHTT proteotoxicity through a novel mechanism. Thus, the distinct characteristics of amorphous deposits suggest FKBP12 may have altered the aggregation energy landscape of mHTT, leading to formation of completely different conformational polymorphisms in the oligomer and aggregation states (Fig. 6). Though we may rule out this alteration is neither driven by the PPIase activity nor the coaggregaton process, the detailed mechanism require further investigation.

In addition, while perturbed calcium homeostasis has been shown to be a striking characteristic of HD progression *in vivo*, it has also been indicated that abnormal calcium leakage caused by the ryanodine receptor (RyR) is associated with mHTT-induced neural death^41^. As post-translational modifications, such as hyperphosphorylation and oxidation, may change the role of RyR in calcium regulation, it is possible that the proteotoxic mHTT species may have direct effects on RyR^43^. As our findings demonstrate that mHTT can be transformed into benign species by FKBP12, it would be interesting to investigate the effect of benign mHTT on calcium regulation in future studies.

In summary, our data suggest that FKBP12 acts as a modifier to regulate the proteotoxicity of mHTT, exhibits significant beneficial effects on the survival and neurite outgrowth of a neuronal cell line, and confers neuroprotection in *C. elegans.* Instead of inhibiting aggregate production, FKBP12 structurally promotes the formation of benign amorphous aggregates and interrupts the oligomerization process to form low-toxic intermediates. Our findings suggest a novel function of FKBP12 and its possible contribution to the pathogenesis of HD as well as other neurodegenerative diseases (e.g. polyQ disease and ALS). Given that the amount of FKBP12 protein is reduced in several important diseases, further investigation of the mechanism underlying the reduction of FKBP12 may facilitate the future development of therapeutic interventions for neurodegenerative diseases.

## Materials and Methods

### Constructs

Glutathione S-transferase (GST)-Htt(Q)_25_ and GST-Htt(Q)_43_ were prepared by subcloning the Htt exon 1 with the applicable polyglutamine tract from pcDNA3.1-Htt-(Q)_25_-hrGFP construct or Htt-(Q)_43-_DsRed, respectively, into the pGEX4T-1 vector (GE Healthcare). Histidine (His) tags were added to the C-termini of both constructs. To generate pET21a-FKBP12-His, a cDNA fragment encoding FKBP12 was amplified by PCR from the pcDNA3.1-FKBP12–V5 construct and ligated into the pET21a-His vector. To generate Htt(Q)_25_-eYFP and Htt(Q)_109_-eYFP, Htt exon 1 containing polyglutamine tracts of different lengths were subcloned from pcDNA3.1-Htt-(Q)_25_-hrGFP and pcDNA3.1-Htt-(Q)_109_-hrGFP constructs into pcDNA3 vector (Invitrogen Life Technologies). Enhanced YFP fluorescence (eYFP) protein was subsequently fused at the C-terminus of each construct. For *C. elegans* experiment, pan-neuronally expressing vector punc-119::mCherry was kindly provided by Dr. Chun-Liang Pan (National Taiwan University). punc-119::FKBP12::mCherry was created by ligating PCR-amplified FKBP12 fragment with XmaI and AgeI sites. All of the constructs were verified and confirmed by DNA sequencing.

### Thioflavin T fluorescence assay

GST-Htt(Q)_25_, GST-Htt(Q)_43_ and FKBP12 proteins were purified and determined the concentrations and purity by SDS-PAGE staining with Coomassie blue and Bradford assay, respectively (details in Supplementary Materials and Methods). The amyloid properties of HTT proteins harboring different polyglutamine expansions were monitored using the Thioflavin T (ThT) fluorescence assay, as described previously^29^. GST-Htt(Q)_n_ (n = 25 or 43) at a concentration of 3 μM was treated with thrombin, and incubated in the presence or absence of 3 μM FKBP12 for 1 or 7 days. At the indicated time, ThT dye (Sigma) was applied to a final concentration of 100 μM. Fluorescence measurements were obtained at an excitation wavelength of 442 nm, and the emission spectra from 460 to 600 nm were recorded with a Hitachi F4500 Fluorescence Spectrometer. For determining the amyloid properties of the sorted materials from the P1 fraction, 4 × 10^6^ particles were collected and centrifuged at 14 K rpm for 30 min at 4 °C. Pellets were resuspended with 80 μl ddH_2_O and then stained with ThT dye; the resulting fluorescence spectra were measured as described above. Values are shown as means ± Standard Error of the Mean (SEM) calculated from three independent experiments.

### Fluorescence-activated cell sorting (FACS)

In brief, transfected N2A cells were lysed with ice cold RIPA buffer containing freshly added complete protease inhibitor cocktail (Roche) and Benzonase Nuclease (Merck Millipore), mixed with 2% SDS, and then incubated on ice for 30 minutes with rotation. Cell lysates were harvested and loaded into a FACSAria cell sorter (BD Biosciences). The flow rate was set at 4,000 events/second, and the gating criteria were established based on the fluorescence intensity of eYFP. The histograms of the N2A cell lysate, Htt(Q)_25_-eYFP, and Htt(Q)_109_-eYFP enable effective gating for each of the fractions. The purity of the sorted materials was reanalyzed for 2,000 events, and the percentages of particles that fall into the appropriate gate were calculated. The collected materials from P1 were centrifuged at 14 K rpm for 30 min at 4 °C. P2 and P3 were ultracentrifuged in a MLS-50 rotor (Beckman Coulter) at 40K rpm for 16 hours at 4 °C. All of the specimens were resuspended with 80 μl ddH_2_O for further experiments.

### *In vivo* DSP-crosslinking and co-immunoprecipitation assay

N2A cells were co-transfected with pcDNA3-Htt(Q)_25_-eYFP or pcDNA3-Htt(Q)_109_-eYFP and either pcDNA3 or pcDNA3.1-FKBP12-V5. After 39 hours, cells were washed twice with PBS and incubated with 1 mM dithiobis(succinimidyl propionate) (DSP) (Pierce, Thermo Scientific) for 40 minutes at room temperature. The reaction was quenched with DSP quenching solution (50 mM Tris-HCl, pH 7.5) for 30 min at room temperature, and cells were then washed twice with PBS. Following lysis (induced by 10 passages through a 24-gauge needle in PBS buffer containing protease inhibitor cocktail), cell lysates were centrifuged at 14 K rpm for 10 min at 4 °C, and supernatants were collected. For the co-immunoprecipitation assay, 250 μg of total protein in each sample were incubated with 1.25 μl V5 antibody and 20 μl Protein G Dynabeads (Invitrogen) for 4.5 hours at 4 °C with rotation. Beads were washed twice with PBS buffer containing 0.1% Tween 20. Extracted proteins and immunocomplexes were eluted and reverse cross-linked in 5X Laemmli sample buffer supplemented with DTT at 95 °C. Samples were resolved by SDS-polyacrylamide gel electrophoresis (PAGE) and detected by Western blot using V5 antibody (1:5000; Invitrogen) and 1C2 antibody (1:2500; Millipore).

### Size exclusion chromatography (SEC) and slot blot assay

N2A cells were co-transfected with pcDNA3-Htt(Q)_25_-eYFP or pcDNA3-Htt(Q)_109_-eYFP and either pcDNA3 vector or pcDNA3.1-FKBP12–V5. After 48 hours, the transfected cells were harvested in 500 μl of ice-cooled PBS buffer containing protease inhibitor cocktail (Roche) and Benzonase Nuclease (Merck Millipore), and sonicated on ice for 1 min. Extracts were centrifuged at 14 K rpm for 30 min at 4 °C, and concentrations were determined using the BCA assay. Samples containing 120 μg of total proteins in a volume of 500 μl were filtered with a 0.22 μm filter (Millipore) and fractionated with a Superdex 200 10/300 column (GE Healthcare) using a flow rate of 0.3 ml/min. Each fraction (1 ml volume/fraction) was collected and subjected to Western blot and slot blot analysis. Htt oligomeric species in each fraction were quantified by densitometry (Image J). The percentage of Htt species was calculated by dividing the density of each fraction by the summed density of every fraction (fractions 7–18). For slot-blotting analysis, the collected fractions were applied to a 0.45 μm nitrocellulose membrane (Schleicher & Schuell) and probed with an A11 antibody (1:1000, Invitrogen) overnight at 4 °C, followed by incubation with HRP-conjugated anti-rabbit secondary antibody (1:7500; Jackson ImmunoResearch) for 1 hour at room temperature. Blots were detected using an ECL chemiluminescent kit.

### Cell viability and neurite outgrowth assay

For the viability assay, 10^5^ N2A cells were seeded in a 12-well plate overnight, and then co-transfected with the indicated constructs. At 45 hours after transfection, cells were exposed to 100 μM H_2_O_2_ for 3 hours, and cell viability was then determined using the AlamarBlue (AbD Serotec) assay, as described previously^29^. The cell viability ratio was calculated as follows: cell viability = (sample-background)/(PBS treatment-background).

For the neurite outgrowth assay, transfected N2A cells were induced to differentiate by incubation in culture media containing 10 μM retinoic acid (Sigma) and 1% FBS for 48 hours. Phase contrast and fluorescent images of the differentiated cells were captured using a Nikon eclipse TiE & EMCCD: Andor 888. Only eYFP positive cells with intact nuclei and normal shape/volume were chosen for analysis. Of these cells, only those with processes twice as long as their cell body were considered to be neurite-positive cells. Over 100 cells per sample were counted, and the experiments were performed in triplicate. For the cell viability and nerite outgrowth assays, the error bars represent the Standard Deviation (SD) of three independent experiments.

### *C. elegans* strains maintenance and behavioral assays

The n(Q)_19_ (AM49 rmIs172 [F25B3.3p::Q19::CFP]) and n(Q)_67_ strains (AM44 rmIs190 [F25B3.3p::Q67::CFP]) were requested from Caenorhabditis Genetic Center (CGC)^24^. The strains of nematodes were maintained with standard procedure and grown at 20 °C as previously described^44^. To generate the transgenic animal, 50 ng/μl punc-119::FKBP12::mCherry, 10 ng/μl myo-2::GFP and 100 ng/μl pcDNA3.1 were co-injected to wild-type N2 hermaphrodite as described previously^45^. Meanwhile, transgenic line bearing with punc-119::mCherry was also created as control. At least 3 independent lines were obtained, respectively and the healthiest lines were chosen for further experiments. For all assays followed, the transgenic young hermaphrodite adults (post-L4 20–24 hours) were used. For monitoring the body size, the young adults were immobilized and the corresponding images were captured and measured with Olympus BX-53 upright microscope. For motility assay, the body bends of the corresponding in duration of 30 seconds of the various strains were documented through SMZ800N stereomicroscope equipped with a CCD camera (Nikon). A body bend was counted as the head of the animal travels across the mid-body of the animal in M9 buffer^24^,^46^. For monitoring transgenic expressivities, the confocal images were captured with LSM 780 (Carl Zeiss). All experiments were conducted under blind conditions.

### Peptide preparation and identification

K_2_Q_40_K_2_ peptide was requested from the CEM Corporation. G295S was synthesized by the batch Fmoc polyamide method using the peptide synthesizer (PS3, Rainin Instrument). Rink amide AM resin was applied as the solid support. Both of the crude peptides were purified and confirmed by high-performance liquid chromatography (HPLC, Agilent). The molecular weights of the peptides were identified by Matrix-Assisted Laser Desorption/Ionization (MALDI) mass spectroscopy. K_2_Q_40_K_2_ was disaggregated by incubating in a 1:1 mixture of trifluoroacetic acid (TFA; Alfa Aesar) and hexafluoroisopropanol (HFIP; Matrix Scientific) to dissolve the preexistent aggregates as described earlier^47^. Solvent was evaporated by nitrogen gas and the disaggregated peptide was stored at −80 °C. Before experiment, PBS was added to K_2_Q_40_K_2_ to initiate the fibrillization process.

### Statistical analysis

Statistical analysis was performed using one-way analysis of variance followed by posthoc Tukey’s test or Student’s *t*-test. Significance was accepted at p < 0.05.

## Additional Information

**How to cite this article**: Sun, C.-S. *et al.* Conformational switch of polyglutamine-expanded huntingtin into benign aggregates leads to neuroprotective effect. *Sci. Rep.* **5**, 14992; doi: 10.1038/srep14992 (2015).

## Supplementary Material

Supplementary Information

Supplementary Information
