# Supplementary material for: Conformational switch of polyglutamine-expanded huntingtin into benign aggregates leads to neuroprotective effect
Source: Sci Rep. 2015 Oct 9;5:14992. doi: 10.1038/srep14992 (PMC4598856; doi:10.1038/srep14992)
Supplement: Supplementary Information [file srep14992-s1.pdf]

Supplementary information

Conformational switch of polyglutamine-expanded huntingtin into benign aggregates leads to neuroprotective effect

Chia-Sui Sun[1,2,3,4], Chi-Chang Lee[2], Yi-Ni Li[2], Sunny Yao-Chen Yang[2], Chih-Hsiang Lin[2], Yi-Che Chang[2], Po-Fan Liu[2], Ruei-Yu He[2], Chih-Hsien Wang[5], Wenlung Chen[5], Yijuang Chern[3*], Joseph Jen-Tse Huang[2*]

[1]Taiwan International Graduate Program in Molecular Medicine, National Yang-Ming University and Academia Sinica, Taipei, Taiwan, [2]Institute of Chemistry and [3]Institute of Biomedical Sciences, Academia Sinica, Taipei, Taiwan, [4]Institute of Biochemistry and Molecular Biology, National Yang-Ming University, Taipei, Taiwan, [5]Department of Applied Chemistry, National Chiayi University, Chiayi, Taiwan,

*To whom correspondence should be addressed:
Joseph Jen-Tse Huang, Institute of Chemistry, Academia Sinica: No.128, Sec. 2, Academia Road, Nankang, Taipei 11529, Taiwan. Tel: 886-2-27898652; Fax: 886-2-27831237; E-Mail: jthuang@gate.sinica.edu.tw

Yijuang Chern, Institute of Biomedical Sciences, Academia Sinica: No.128, Sec. 2, Academia Road, Nankang, Taipei 11529, Taiwan. Tel: 886-2-27829028, Fax: 886-2-27829143; E-mail: bmychern@ibms.sinica.edu.tw

Supplementary Information:

Part 1 - Supplementary Figures 1-8
Part 2 - Supplementary Video Legend
Part 3 - Supplementary Materials and Methods

Part 1- Supplementary Figures

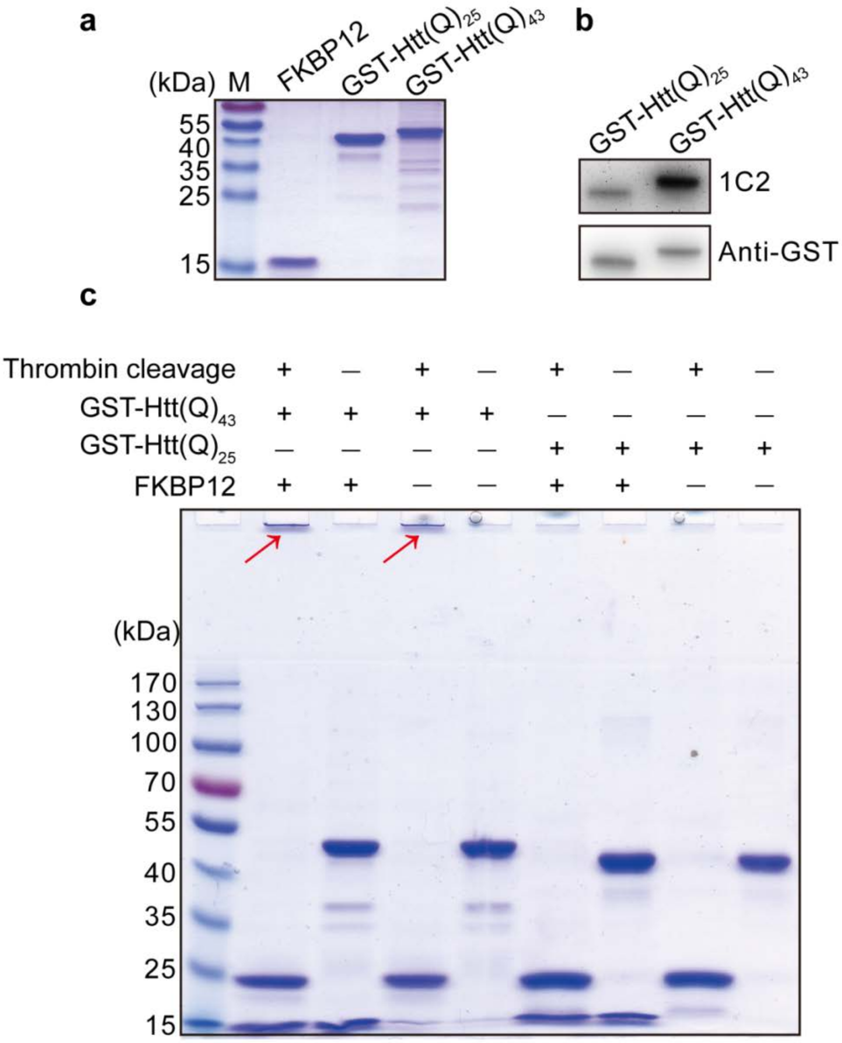

Supplementary Figure 1. The removal of GST by triggered the formation of high-molecular weight SDS-insoluble aggregates in Htt(Q)$_{43}$. The purity of the recombinant proteins were subjected to SDS-PAGE **(a)** and analyzed by western blot using anti-GST and 1C2 antibodies **(b)**. **(c)** Thrombin was treated to liberate GST tag in GST-Htt(Q)$_{25}$ or GST-Htt(Q)$_{43}$ group in the presence/absence of FKBP12 for 24 hr. Htt(Q)$_{43}$, but not Htt(Q)$_{25}$, formed high-molecular weight (MW), SDS-insoluble aggregates accumulated in the stacking gel (red arrow). Co-incubation of FKBP12 with Htt(Q)$_{43}$ also resulted in massive accumulation of aggregates (red arrow). However, none of the aforementioned phenomenon can be seen in Htt(Q)$_{25}$ group.

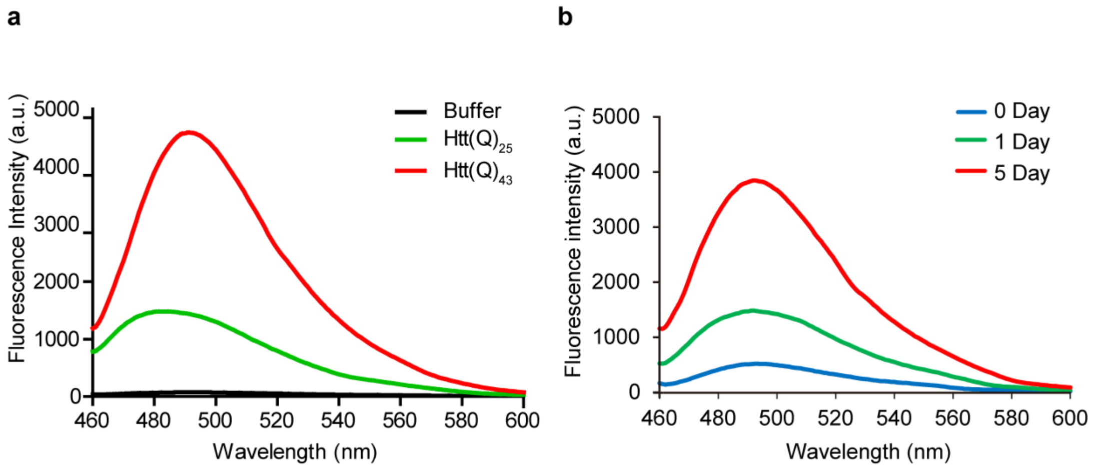

Supplementary Figure 2. Htt(Q)$_{43}$ exhibits high amyloid property and elevated amyloidogenicity can also be observed over time. **(a)** The ThT fluorescence of Htt(Q)$_{25}$ and Htt(Q)$_{43}$ after GST removal. Green, red, and black line corresponds to Htt(Q)$_{25}$, Htt(Q)$_{43}$, and ThT buffer control respectively. **(b)** The amyloidogenicity of Htt(Q)$_{43}$ was monitored by ThT assay at various time points. Blue, green and red represents the ThT fluorescence at day 0, 1, and 5 after GST removal.

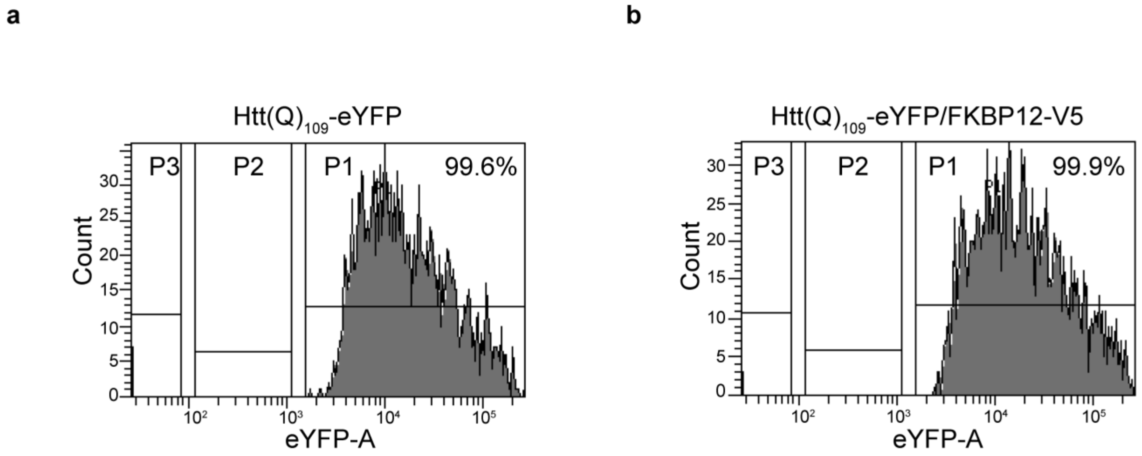

Supplementary Figure 3. The sorted particles from P1 fraction exhibits high purity. (a-b) The sorted inclusion bodies from the P1 fraction were re-analyzed by flow cytometry to check their purity. 99.6% and 99.9% of the sorted particles were eYFP-positive in Htt(Q)$_{109}$-eYFP and Htt(Q)$_{109}$-eYFP/FKBP12 group, respectively.

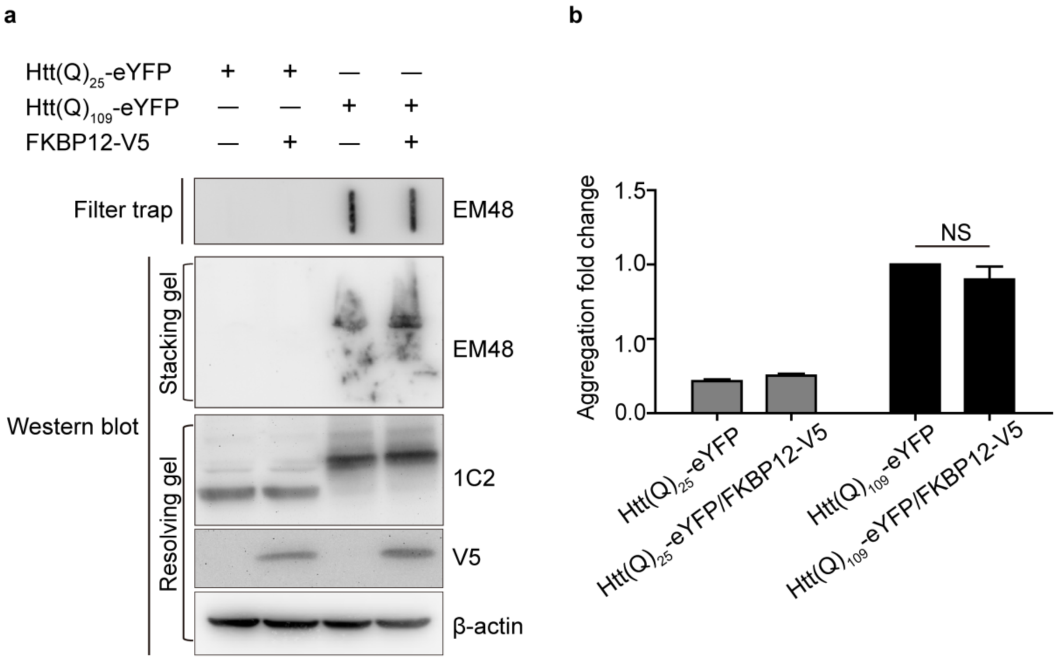

Supplementary Figure 4. The levels of soluble and insoluble mHTT are not altered when co-expressed with FKBP12. (a) Aggregation analysis of the Htt(Q)$_{n}$-eYFP (n=25, 109) proteins in the presence/absence of FKBP12 were determined by filter trap assay and Western blot. Filter trap assay showed mHTT aggregates were retained on the cellulose membrane as probed by EM48 antibody. Western blot analysis demonstrated accumulation of high MW insoluble mHTT aggregates at the stacking gel in both Htt(Q)$_{109}$-eYFP and Htt(Q)$_{109}$-eYFP/FKBP12 group. Soluble Htt(Q)$_{25}$-eYFP, Htt(Q)$_{109}$-eYFP, FKBP12-V5, and actin were probed by 1C2, V5, and β-actin antibody, respectively. **(b)** Quantified results of the mHTT aggregates compared with Htt(Q)$_{109}$-eYFP only. The overexpression of FKBP12 did not change the aggregation level of mHTT. Results were means ± SEM of three independent experiments (NS = not significant).

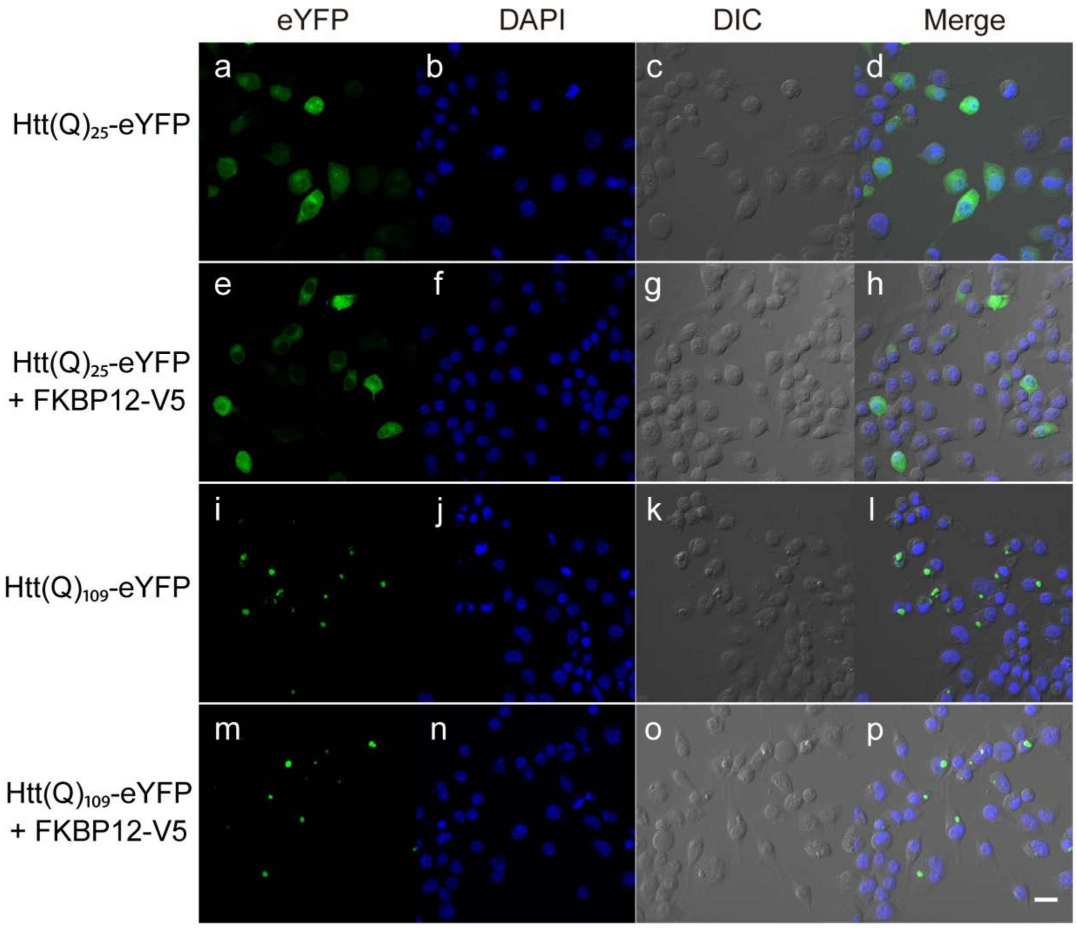

Supplementary Figure 5. Overexpression of FKBP12 did not change the localization of Htt(Q)$_{25}$-eYFP and Htt(Q)$_{109}$-eYFP in N2a cell. The confocal microscopy images of the eYFP-fused Htt(Q)$_n$ constructs (n=25, 109) co-transfected with FKBP12 or vector as visualized by confocal microscopy. After 48 hrs, Htt(Q)25-eYFP co-expressed with vector or FKBP12 displayed diffused eYFP signal in the cytoplasm. As for Htt(Q)$_{109}$-eYFP, overexpression with FKBP12 or vector both showed formation of fluorescent inclusions in the cytoplasmic area. Bar indicates 20 μm.

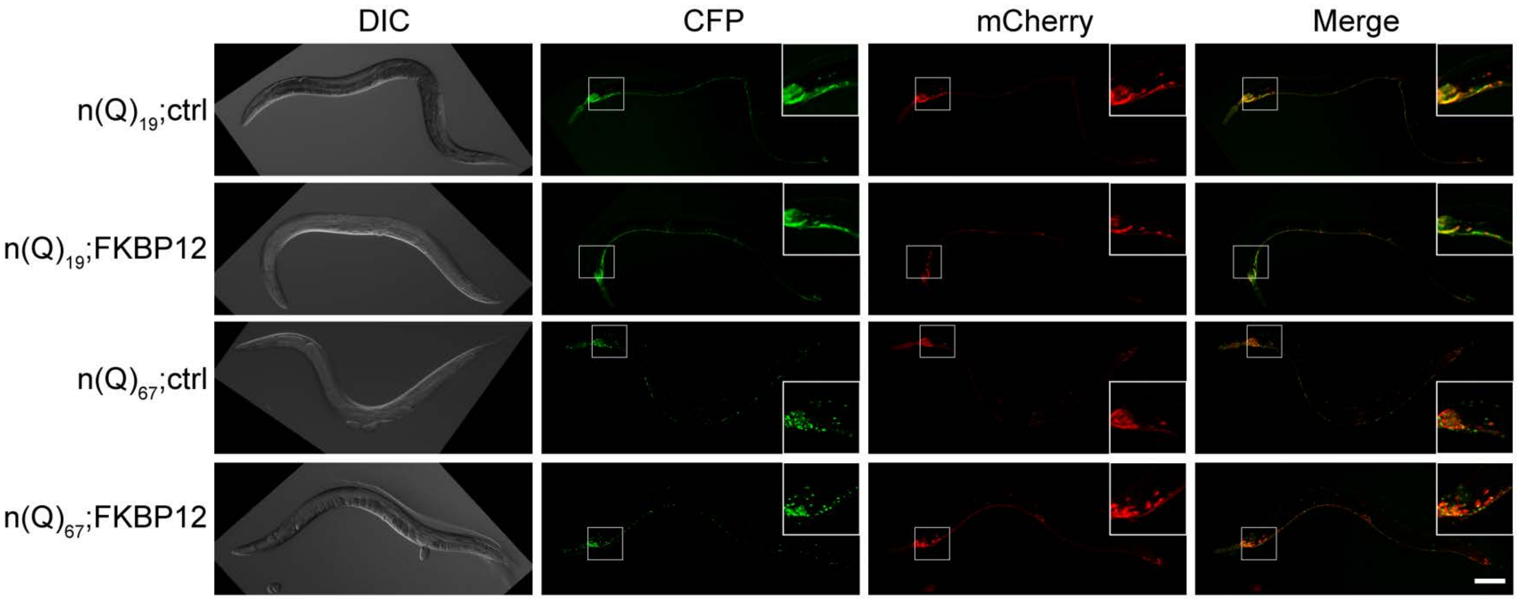

Supplementary Figure 6. Micrographs of the pan-neuronal polyQ proteins with or without the co-expression of FKBP12 in *C. elegans*. CFP and mCherry were reporters as indicatives of the polyQ and FKBP12 expressivities, respectively. The anterior is to the left and the ventral side is to the bottom. The insets in n(Q)$_{19}$;FKBP12 group were rotated 90° counterclockwise for easier observation. Insets showed enlarged fluorescence micrographs as diffused, soluble fluorescence pattern were displayed in both n(Q)$_{19}$;ctrl and n(Q)$_{19}$;FKBP12 animals. Conversely, n(Q)$_{67}$;ctrl and n(Q)$_{67}$;FKBP12 animals exhibited distribution of fluorescent foci. Note that the distribution of the polyQ puncta in n(Q)$_{67}$;ctrl and n(Q)$_{67}$;FKBP12 animals were similar. Each of the chosen animals were young adult (post-late L4 20-24 hr) transgenic worms. Scale bar represents 100 μm.

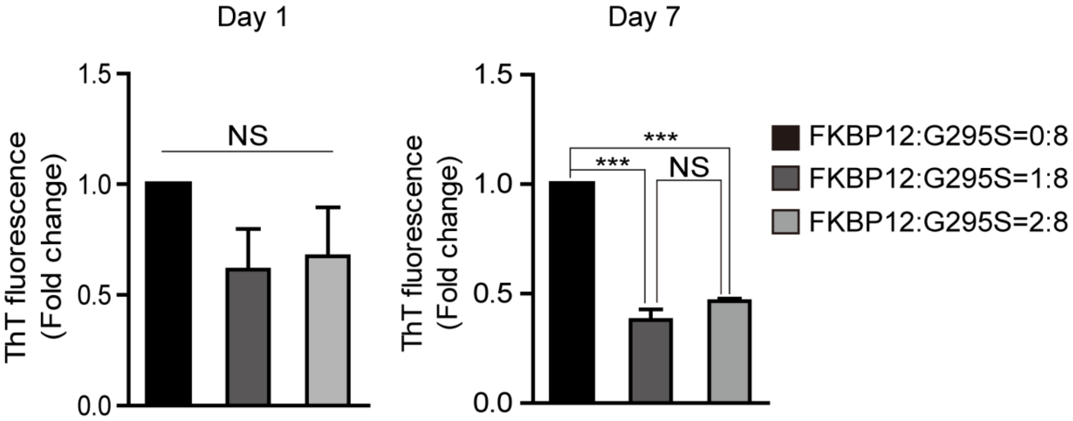

Supplementary Figure 7. FKBP12 decreases the amyloidogenicity of G295S peptide. FKBP12 was co-incubated with G295S at molar ratios of 0:8, 1:8, and 2:8 for 1 to 7 days. At day 7, co-incubation of FKBP12 with G295S (1:8 and 2:8 molar ratios) showed significant decreased ThT fluorescence intensity. Results were means ± SEM of three independent experiments (***$p<0.001$, NS = not significant).

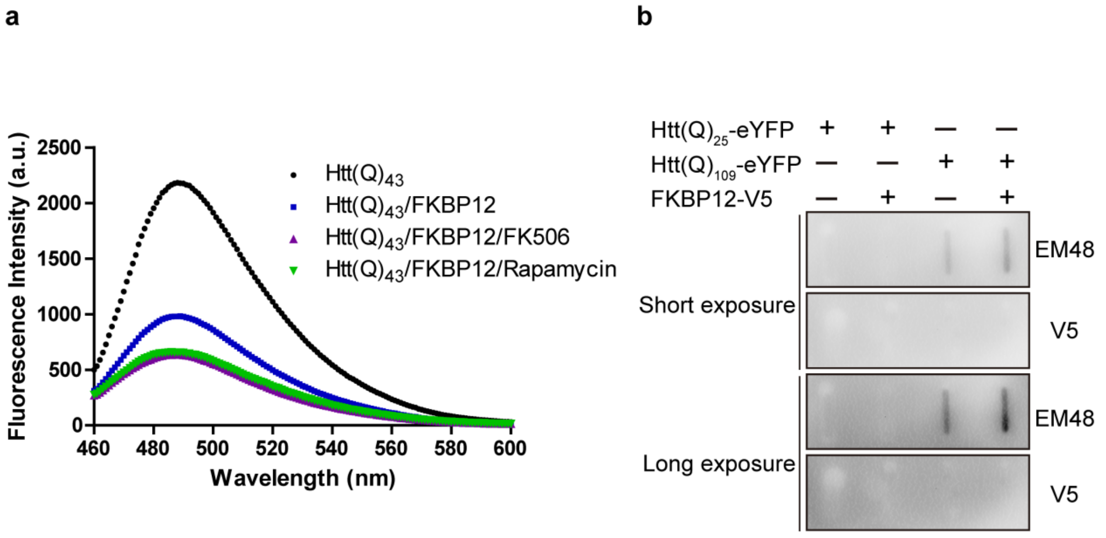

Supplementary Figure 8. FKBP12 does not modulate mHTT amyloidogenicity by its PPIase activity or the coaggregation with mHTT. After GST removal for 24 hours, the ThT fluorescence of Htt(Q)$_{43}$ and Htt(Q)$_{43}$/FKBP12 in the presence/absence of different PPIase inhibitors, FK506 and Rapamycin, are shown in **(a)**. FKBP12 suppressed the amyloidogenicity of Htt(Q)$_{43}$ in the presence of FK506 (Htt(Q)$_{43}$/FKBP12/FK506) or Rapamycin (Htt(Q)$_{43}$/FKBP12/ Rapamycin). **(b)** The coaggregation analysis of Htt(Q)$_n$-eYFP (n=25, 109) coexpressed with/without FKBP12-V5 in N2a cells were determined by filter trap assay. The presence of mHTT and FKBP12 protein in the insoluble aggregates were probed with EM48 and V5 antibodies, respectively. FKBP12 cannot be detected either at short or long exposure times.

Part 2 - Supplementary video

Supplementary video 1. FKBP12 reduced polyQ-mediated motility impairment in *C. elegans*. The footage revealed the motilities of various *C. elegans* strains by monitoring their swimming pattern in M9 buffer. Note that $n(Q)_{67}$;ctrl animals swam reluctantly than $n(Q)_{19}$;ctrl and $n(Q)_{19}$;FKBP12 animals. On the contrary, the swimming rate of $n(Q)_{67}$;FKBP12 animal was significantly improved than that of $n(Q)_{67}$;ctrl animals. Clockwise from the left-up panel: $n(Q)_{19}$;ctrl, $n(Q)_{19}$;FKBP12, $n(Q)_{67}$;ctrl, and $n(Q)_{67}$;FKBP12.

Part 3 - Supplementary Materials and Methods

Protein expression and purification

Both the GST-Htt(Q)$_{25}$ and GST-Htt(Q)$_{43}$ constructs were individually transformed into *E. coli* SCS1 (Stratagene). GST-Htt(Q)$_{25}$ was induced at 20℃ overnight using 0.1 mM isopropyl 1-thio-*β*-D-galactopyranoside (IPTG). The induced cells were pelleted and lysed in Buffer A (50 mM sodium phosphate, pH 8, 150 mM NaCl, 1 mM EDTA, 5 mM DTT, 1 mM PMSF, 10% glycerol). Soluble protein was loaded onto an Ni-NTA column, and the bound proteins were eluted with Buffer B (50 mM sodium phosphate, pH 8, 300 mM NaCl, 1 mM EDTA, 5 mM DTT, 1 mM PMSF, 10% glycerol) containing 200 mM imidazole. For secondary purification, the collected eluents were further incubated with 50% slurry of Glutathione Sepharose 4B (GE Healthcare) equilibrated with Buffer A at 4℃ overnight. Bound GST-tagged protein was eluted with Buffer A containing 30 mM reduced glutathione. Purified GST-Htt(Q)$_{25}$ was dialyzed against thrombin cleavage buffer (50 mM Tris-HCl, pH 8, 150 mM NaCl, 2.5 mM $CaCl_2$, 0.1% β-mercaptoethanol). To prepare GST-Htt(Q)$_{43}$, protein was induced at 25℃ overnight by the addition of 1 mM IPTG. The induced cells were pelleted and lysed in Buffer A containing 6 M GdnCl. Soluble GST-Htt(Q)$_{43}$ was then added to an Ni-NTA column, and eluted with 250 mM imidazole. The eluents were dialyzed against thrombin cleavage buffer with decreasing amounts of GdnCl (5, 4, 3, 2, 1, 0 mM) in a stepwise manner. The purity and concentration of GST-Htt(Q)$_{25}$ and GST-Htt(Q)$_{43}$ were determined by 12% SDS-PAGE staining with Coomassie blue and Bradford assay, respectively.

For the preparation of FKBP12 proteins, BL21 competent cells were transformed with pET21a-FKBP12-His, and protein expression was induced at 37℃ overnight using 2mM IPTG. Cells were pelleted, resuspended, and lysed in binding buffer (20 mM sodium phosphate, 500 mM NaCl, pH 7). Soluble FKBP12 was purified using a Ni-NTA column and size exclusion chromatography (Superdex 200 10/300; GE Healthcare). The purity and concentration of FKBP12 were determined by SDS-PAGE staining with Coomassie blue and Bradford assay, respectively.

In vitro GST-Htt(Q)$_{\mathbf{n}}$ aggregation

GST-Htt(Q)$_{\mathbf{n}}$ (n=25 or 43) at a concentration of 3 μM was treated with thrombin protease (GE Healthcare), in the presence or absence of 3 μM FKBP12, at 22℃ with vigorous shaking at 1400 rpm (Eppendorf Thermomixer) to remove the GST moiety. At the indicated time, the reaction was terminated by the addition of the equivalent amount of 100 mM DTT and 4% SDS, followed by heating at 98℃ for 10 min. Samples were assessed for further experiments.

SDS-PAGE and Western blot

Transfected N2A cells were extracted with ice-cold RIPA buffer (50 mM Tris-HCl pH 7.6, 150 mM NaCl, 1 mM EDTA, 0.5% sodium deoxycholate, 1% NP-40, 0.1% SDS) containing 2% SDS, protease inhibitor cocktail (Roche), and Benzonase Nuclease (Merck Millipore). Cell lysates were spun at 14K rpm for 30 min at 4℃, and supernatants were then collected. Samples were resolved by 10% SDS-PAGE, electrotransferred to a polyvinylidene difluoride membrane (Millipore), and probed with primary and secondary antibodies. The immunoreactive signals were detected using an ECL chemiluminescent kit (Thermo Fisher Scientific), and the intensities of individual bands were quantified using ImageJ software.

Transmission electron microscopy (TEM) and immunogold labeling TEM analysis

Samples from *in vitro* GST-Htt(Q)$_n$ aggregation reaction and fluorescence-activated cell sorting (FACS) were collected as described. Five microliter aliquots of sample was taken and allowed to absorb to 300 mesh glow-charged, formvar- and carbon-coated copper grids for 4 minutes. Each sample was then negatively stained with 2% (w/v) uranyl acetate for 50 sec. Grids were viewed with a Hitachi H-7000 transmission electron microscope at 75 kV. For immunogold labeling TEM analysis, samples were blocked in 1% BSA in PBS for 1 hour, followed by incubation with EM48 (1:20) at 4℃ overnight. Following three rinses with PBS, samples were probed with 12 nm colloidal gold anti-mouse secondary antibody (1:30; Jackson ImmunoResearch) for 1 hour at room temperature. After rinsing with PBS, samples were fixed with 1% glutaraldehyde in PBS for 10 min, followed by 2% uranyl acetate staining for 70 sec, and observation under a Hitachi H-7000 transmission electron microscope.

Filter trap assay

To assess the effect of FKBP12 on Htt aggregation, N2A cells were transiently co-transfected with Htt(Q)$_n$-eYFP (n=25, 109) and pcDNA3-FKBP12-V5. After 48h, transfected N2A cells were lysed in RIPA buffer containing 2% SDS, protease inhibitor cocktail (Roche), and Benzonase Nuclease (Merck Millipore). Cell lysates were collected and filtered through 0.22 μm cellulose acetate (Schleicher & Schuell), washed twice with 0.1% SDS, and blocked in blocking solution (3% BSA w/v; 0.1% Triton X-100 in PBS). Htt(Q)$_n$-eYFP aggregates were probed with a Huntingtin-specific antibody, EM48 (1:500; Merck Millipore), as primary antibody, followed by incubation with anti-mouse secondary antibody conjugated to horseradish

peroxidase (HRP) (1:7500; Jackson ImmunoResearch). The reaction was developed using an ECL chemiluminescent kit (Thermo Fisher Scientific), and signals corresponding to SDS-insoluble aggregates were quantified with Image J software. Values are shown as means ± Standard Error of the Mean (SEM) calculated from three independent experiments.

Cell culture and transfection

N2A cells were maintained in Dulbecco's Modified Eagle Medium supplemented with 10% fetal bovine serum (FBS) (all from HyClone) and antibiotics (100 units/ml penicillin and 100 μg/ml streptomycin; Gibco Invitrogen) in a 37°C incubator with 5% CO_2. N2A cells were co-transfected with 1 μg Htt$(Q)_n$-eYFP (n=25, 109) and either 2 μg pcDNA3 vector or pcDNA3.1-FKBP12-V5, using the TurboFect Transfection Reagent (Thermo Scientific) according to the manufacturer's protocol. After 48 hours, cells were harvested for further experiments.
